# Quality assurance for point-of-care testing in Zimbabwe

**DOI:** 10.4102/ajlm.v5i2.448

**Published:** 2016-10-17

**Authors:** Sibongile Zimuto, Agrippa Mtambara, Ben Cheng, Brad Cunningham, Rodney Taruvinga, Debrah I. Boeras, Raiva Simbi

**Affiliations:** 1 Zimbabwe National Quality Assurance Programme (ZINQAP), Harare, Zimbabwe; 2 Ministry of Health and Child Care, Harare, Zimbabwe; 3 International Diagnostics Centre, London School of Hygiene & Tropical Medicine, London, United Kingdom; 4 SystemOne, Johannesburg, South Africa

## HIV situation in Zimbabwe

**FIGURE 1:**
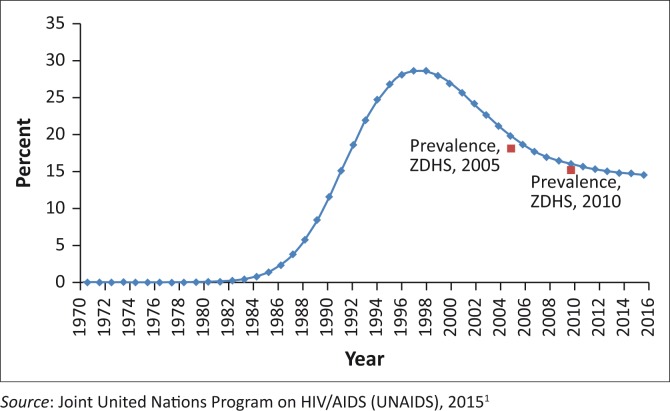
HIV prevalence in Zimbabwe 1970–2016.

Zimbabwe was one of the sub-Saharan countries most severely affected by HIV and AIDS. The first AIDS case was reported in 1985. HIV prevalence increased sharply from 1985 to the mid-90s, peaking at 27.7% in 1997; thereafter, it started to decline (Figure 1).^1^ According to the National AIDS Council, HIV prevalence was estimated to be 15.0% as of the end of 2014.^2^ Zimbabwe has a population of 15.25 million and has a generalised, feminised and homogenous HIV epidemic which continues to decline in terms of new infection rates, prevalence and AIDS-related mortality^1^. However, there are localised areas (11 districts) of high HIV transmission, described as hot spots, which include border districts, growth points, small-scale mining areas, fishing camps and commercial farming settlements.

The number of people living with HIV in Zimbabwe is estimated to be 1 390 211, with an incidence of 0.98, for the 15–49-year age group (Table 1).^1^ New infections are estimated to be 69 105 and annual HIV deaths 63 853; 905 368 people are in of need antiretroviral therapy. Nearly 80.0% of adults have access to antiretroviral therapy, while only 40.5% of children have access to treatment.

**TABLE 1:** Zimbabwe HIV country statistics

Characteristic	No.
Country population	15.25 million
New HIV infections	69 105
People living with HIV/AIDS: HIV+ pregnant women	1 390 211
HIV-positive babies born to HIV-positive mothers	57%
Conventional CD4 machines	138
POC CD4 machines	360
Centralised EID testing sites	3

POC, point of care; EID, early infant diagnosis.

## Laboratory infrastructure for HIV-related testing in Zimbabwe

**FIGURE 2:**
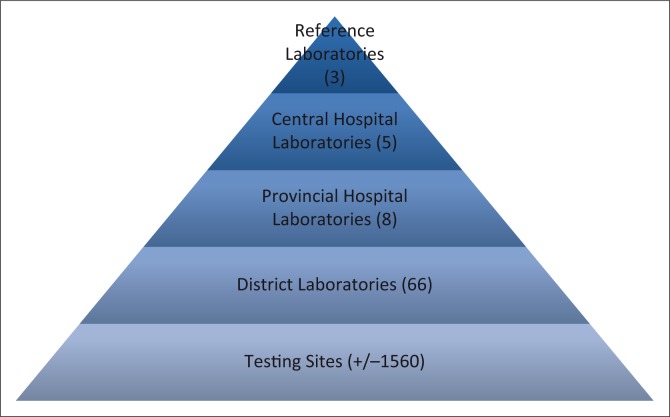
Zimbabwe’s tiered laboratory network.

Zimbabwe has a tiered laboratory network comprising three reference laboratories, five central hospital laboratories, eight provincial hospital laboratories and 66 district laboratories (Figure 2). According to the Ministry of Health and Child Care AIDS and Tuberculosis Unit, there are 4291 HIV rapid testing sites, which may be voluntary counselling and testing or prevention of mother-to-child-transmission of HIV sites. These sites are located at various levels of the Zimbabwe health delivery system. CD4 tests are conducted using conventional equipment as well as point-of-care (POC) devices. There are 138 conventional CD4 analysers and 360 operational CD4 POC testing sites.

Early infant diagnosis (EID) is conducted at three sites, strategically located in different geographical locations to allow easier access to testing. The EID testing sites are in Harare at the National Microbiology Reference Laboratory, in the north of the country, Mpilo Central Hospital Laboratory in the south and Mutare Provincial Hospital Laboratory in the east. Health facilities send their EID samples to any one of the three laboratories, depending on their geographical location. Based on the number of HIV-positive mothers presenting at prevention of mother-to-child transmission screening that have accessed EID, Zimbabwe’s unmet EID needs are quite low.

Zimbabwe has developed a national viral load scale-up plan in support of the 90-90-90 goals.^3^ Zimbabwe is in the process of procuring eight viral load instruments – two bioMérieux NucliSENS^®^ six Abbott *m*2000 instruments – through Global Fund. The Abbott instruments are due for delivery in October 2016. Partners such as the United States Agency for International Development have pledged to support human resources and other accessories. Zimbabwe has recently completed an evaluation of the Cepheid GeneXpert^®^ viral load platform and the data are being analysed. They also evaluated the DRW Samba I platform, but there are reservations with this method and the country would like to evaluate Samba II.

Zimbabwe plans to roll out POC viral load testing at the district level in view of the complexity of the method and the increased workload on clinical health personnel at lower levels of the health system brought on by task shifting. It is envisaged that POC tests will provide access to viral load testing at low-volume sites in remote settings.

Zimbabwe revised its laboratory’s strategic plan in October 2015 (unpublished), detailing the focus areas for laboratory services in Zimbabwe. The strategic plan will run until 2018.

## Quality assurance in Zimbabwe

The Zimbabwe National Quality Assurance Programme (ZINQAP) is the national EQA provider. ZINQAP provides an accredited proficiency testing programme as well as training and mentorship in quality systems. ZINQAP provides its services to both public- and private-sector laboratories on a cost recovery basis. The ZINQAP EQA programme covers all the major disciplines of laboratory medicine, including microbiology, clinical chemistry, immunology, haematology and blood transfusion. On-site support and supervisory visits are conducted to assist poor performers to determine the cause of the poor performance and implement corrective and preventive actions. According to the Zimbabwe Medical Laboratory Guideline, medical laboratories are required to participate in a proficiency testing programme as a pre-requisite for registration.^4^ However, due to funding limitations, this is not yet been fully implemented. The Ministry of Health and Child Care has developed a policy on POC testing, which guides the selection, validation deployment and quality assurance for POC testing. This policy has yet to be approved and distributed.

**FIGURE 3:**
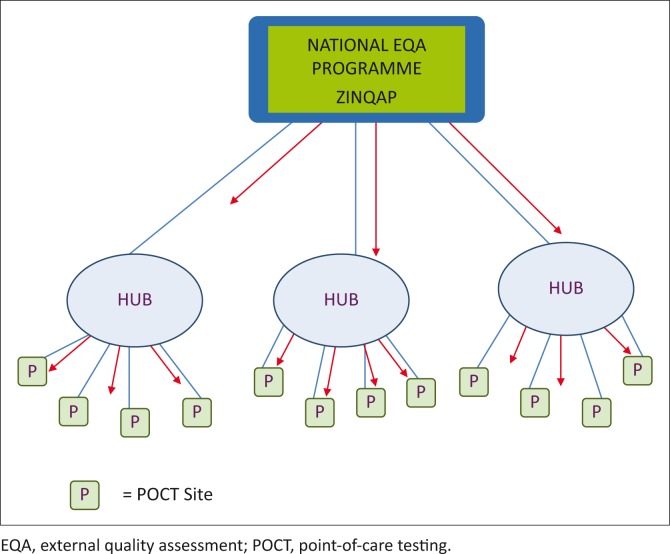
Zimbabwe external quality assessment model.

The EQA implementation models for HIV and related testing are as follows: EQA for HIV rapid testing is composed of proficiency testing panels prepared in-house by ZINQAP and distributed to participating sites on a monthly basis. However, less than 5% of sites are currently on the EQA programme, due to funding limitations. CD4 EQA for conventional CD4 analysers is also run by ZINQAP and is also composed of in-house prepared samples, which are distributed six times a year to participating sites. EQA for CD4 POC testing is prepared by QASI, an initiative of the Public Health Agency of Canada, which was previously distributed by the National Microbiology Reference Laboratory, before being transitioned to ZINQAP. There are 266 POC testing sites on the QASI POC CD4 EQA programme. The London School of Hygiene and Tropical Medicine is conducting a project in Zimbabwe to determine the cost of a national EQA system in collaboration with the Ministry of Health and ZINQAP. This project includes building capacity for ZINQAP to provide EQA to all POC CD4 sites (Figure 3). This model will be used to expand EQA to other POC testing platforms as they are introduced to the country. There are funding constraints for the full roll out of POC EQA. Zimbabwe is engaging all partners and stakeholders to ensure that the future introduction of POC devices includes EQA.

POC testing poses new challenges to EQA, especially taking into account that hundreds of devices are being deployed for use by non-skilled, non-laboratory personnel with limited knowledge of quality practices.^5,6^ EQA for POC testing is an important tool which can provide information on the quality of the whole POC testing process. The connectivity project being piloted in Zimbabwe, which includes a proof of concept for automated reporting of EQA results, presents a possible solution to the EQA challenge for POC testing. Automated connectivity solutions can be used to transmit EQA performance data to the EQA provider for data analysis, so that corrective action can be taken, if necessary, in a timely manner. This overcomes the current problem of a low percentage of sites reporting EQA results. Connectivity can also be perceived as being complementary to EQA, as it not only conveys information on test results but also operational data from the device, which can provide key equipment performance characteristics and quality indicators as part of a continuous quality assurance process. The system can also provide alerts when stocks are low, hence improving supply chain management.

The three EID sites participate in the EQA run by the National Institute for Communicable Diseases, South Africa. The viral load EQA programme is organised by the US Centers of Disease Control and Prevention, Atlanta; currently there is one laboratory site on this programme.

## Existing quality assurance programmes and lessons learnt

Zimbabwe has been participating in EQA programmes since the mid-1980s. Proficiency testing services were obtained from the UKNEQAS, before the establishment of ZINQAP in 1998. Over the years, several lessons have been learnt regarding proficiency testing and EQA. An analysis of proficiency testing participation and performance records provides useful insights that can give guidance for policy makers and implementers. Local ownership of the programme is very important, as a home-grown programme is well versed in local challenges and best placed to come up with interventions to address challenges faced. A locally-coordinated programme also has the advantage of quicker turnaround times and can provide on-site support to poor performers. Local production of panels results in panels with characterisation that is similar to patient samples, which can best assess the testing systems.

Financing of EQA programmes is of critical importance, as they provide important information on the quality of testing and therefore the reliability of the results obtained.^7^ The national HIV response is based on testing; therefore, it is of critical importance to have a system of monitoring the quality of testing. It is important to ensure that there is adequate funding for the initiation and maintenance of the EQA programme. Initial establishment of the EQA programme can be through partner support, however, it is important to ensure the future sustainability and maintenance of the programme with financial support from its beneficiaries. Depending only on partner funding is not sustainable and poses the risk of collapse of the programme when funding is no longer available. It is also very important to have a mechanism that enforces EQA participation, with consequences for sites that do not participate. Clear understanding and appreciation of the value of EQA is critical to sustainability – it is important to establish a culture of quality.

## National quality assurance programme for HIV

A national quality assurance programme for HIV POC testing should provide clear guidance on selection and deployment of test equipment and methods.^8^ There should be a system of validation and/or verification of test methods, equipment and devices before they are put into use. Policies, standard operating procedures and job aides need to be in place to provide the necessary guidance. Training and competency assessments of the personnel conducting the testing are necessary to ensure that tests are carried out correctly and the results obtained are accurate and reliable. An internal quality control system must be in place and documented. A supply chain management system to ensure uninterrupted service delivery is also important. There is also a need to have a proficiency testing programme to independently assess the quality of testing. In addition, it is necessary to have a system to provide on-site support and supervision to poor performers, including root cause analysis, corrective and preventive actions. All testing sites must participate in the programme. A locally-coordinated or run proficiency testing programme is of greater value, as it provides samples that are similar to patient samples. Funding and sustainability of the programme are of critical importance.

Billions of dollars are spent globally on test equipment, reagents and consumables for HIV. The HIV response is based on results obtained from various testing systems and it is thus of critical importance to ensure that there is a system of assessing and confirming that the tests conducted are accurate and reliable, so as to ensure quality patient care and efficient utilisation of resources.

## References

[1] Joint United Nations Programme on HIV/AIDS (UNAIDS) Global AIDS response progress report 2015 [document on the Internet]. c2015 [cited 2016 Sep 27]. Available from: http://www.unaids.org/sites/default/files/country/documents/ZWE_narrative_report_2015.pdf

[2] National AIDS Council (NAC), Zimbabwe HIV & AIDS situation [page on the Internet]. c2011 [cited 2016 Sep 27]. Available from: http://www.nac.org.zw/about/hiv-aids-situation

[3] UNAIDS 90–90–90 – An ambitious treatment target to help end the AIDS epidemic [document on the Internet]. c2014 [cited 2016 Sep 27]. Available from: http://www.unaids.org/en/resources/documents/2014/90-90-90

[4] Ministry of Health & Child Welfare, Zimbabwe Zimbabwe national guidelines on HIV testing and counselling [document on the Internet]. c2005 [cited 2016 Sep 27]. Available from: https://www.k4health.org/sites/default/files/Zimbabwe%20National%20Guidelines%20on%20HIV%20Testing%20and%20Counselling.pdf

[5] Nkengasong JN, Nsubuga P, Nwanyanwu O, et al Laboratory systems and services are critical in global health: time to end the neglect? Am J Clin Pathol. 2010;134(3):368–373. http://dx.doi.org/10.1309/AJCPMPSINQ9BRMU6 20716791 10.1309/AJCPMPSINQ9BRMU6 PMC7109802

[6] Birx D, de Souza M, Nkengasong JN Laboratory challenges in scaling up of HIV, TB and malaria programs: the interaction of health and laboratory systems, clinical research, and service delivery. Am J Clin Pathol. 2009;131(6):849–851. http://dx.doi.org/10.1309/AJCPGH89QDSWFONS 19461092 10.1309/AJCPGH89QDSWFONS

[7] The World Bank Zimbabwe economic update: results-based financing of health clinics helps Zimbabwe to improve service delivery and weather economic headwinds [page on the Internet]. c2016 [cited 2016 Sep 27]. Available from: http://www.worldbank.org/en/country/zimbabwe/publication/zimbabwe-economic-update-changing-growth-patterns-improving-health-outcomes

[8] Zimbabwe Ministry of Health & Child Welfare Zimbabwe national HIV and AIDS strategic plan 2015–2018. Harare [document on the Internet]. c2015 [cited 2016 Sep 27]. http://www.nac.org.zw/sites/default/files/ZNASP%20III%20Final%20(1).pdf

